# Predictive ability of the Mini Nutritional Assessment Short Form (MNA-SF) in a free-living elderly population: a cross-sectional study

**DOI:** 10.7717/peerj.3345

**Published:** 2017-05-18

**Authors:** Raimunda Montejano Lozoya, Nieves Martínez-Alzamora, Gonzalo Clemente Marín, Silamani J.A. Guirao-Goris, Rosa María Ferrer-Diego

**Affiliations:** 1 La Fe School of Nursing, University of Valencia, Valencia, España; 2 Health Research Institute La Fe (IIS La Fe), Valencia, España; 3 Department of Applied Statistics and Operational Research and Quality, Polytechnic University of Valencia, Valencia, España; 4 Department of Nursing, Faculty of Health Sciences, University of Alicante, Alicante, España

**Keywords:** Nutritional assessment, MNA-SF, Free-living elderly

## Abstract

**Background:**

Various scales have been used to perform a quick and first level nutritional assessment, and the MNA is one of the most used and recommended by experts in the elderly in all areas. This scale has a short form, the MNA-SF, revised and validated in 2009, which has two versions: the BMI-MNA-SF contains the first six items of the full scale including Body Mass Index while the CC-MNA-SF includes Calf Circumference instead of BMI.

**Objective:**

To evaluate the predictive ability for nutritional status of the two versions of the MNA-SF against the MNA in free-living elderly in the province of Valencia.

**Methods:**

Cross-sectional study of 660 free-living elderly in the province of Valencia selected in 12 community centres using stratified sampling by blocks. Inclusion criteria: being aged 65 or over, living at home, having functional autonomy, residing in the province of study for more than one year, regularly attending community centres and voluntarily wanting to take part.

**Results:**

Of the 660 subjects studied, 319 were men (48.3%) and 341 (51.7%) women with a mean age of 74.3 years (SD = 6.6). In terms of nutritional assessment, using the BMI-MNA-SF and the CC-MNA-SF we found that 26.5% and 26.2% were at risk of malnutrition and 0.9% and 1.5% were malnourished respectively. With the full MNA, 23.3% were at risk of malnutrition. Spearman’s rank correlation coefficients indicate a high association between the full MNA score and the MNA-SFs scores (BMI-MNA-SF: *ρ* = 0.78*p* < 0.001; CC-MNA-SF: *ρ* = 0.78*p* < 0.001). In addition we obtained a very high correlation between the two MNA-SFs (*ρ* = 0.96*p* < 0.001). We evaluated the agreement between the full MNA and the MNA-SFs classification in three nutritional categories (normal nutritional status, risk of malnutrition, malnutrition) with Cohen’s kappa coefficients (BMI-MNA-SF: *κ* = 0.54*p* < 0.001; CC-MNA-SF: *κ* = 0.52*p* < 0.001). These values indicate moderate agreement with the full MNA. There is very good agreement between the BMI-MNA-SF and CC-MNA-SF (*κ* = 0.88*p* < 0.001). In order to determine the ability of both MNA-SFs to identify subjects not requiring any nutritional intervention, we considered the dichotomised categorisation of the full MNA and the MNA-SFs as “normal nutritional status” vs. “malnutrition and risk of malnutrition” Areas under the ROC curves using MNA as the gold standard indicate moderately high prognostic accuracy (BMI-MNA-SF: *AUC* = 0.88*p* < 0.001; CC-MNA-SF: AUC = 0.87 *p* < 0.001). Both versions of the MNA-SF showed similar sensitivity, specificity and diagnostic effectiveness (BMI-MNA-SF: 73.4%, 86.6%, 83.5%; CC-MNA-SF 73.4%, 86.2%, 83.2%).

**Conclusions:**

In its two versions the MNA-SF presents useful predictive ability against the MNA. The advantage of the CC-MNA-SF is that using it requires fewer resources and less time in primary care, although always the characteristics of the population must take into account to make the right decision based on the MNA-SF scales.

## Introduction

In recent years we have seen unprecedented global demographic change in the ageing of the world population. This social phenomenon will continue to grow and experts forecast that by 2050 the elderly will account for over a third of the total population of some developed countries. At 34.4%, Spain will be the fourth country in the world with the highest percentage of people over 65 (Vidal Domínguez & Fernández Portela, 2014).

The elderly are a highly vulnerable group susceptible to nutritional difficulties (Tena & Serrano, 2002) which if not prevented or treated in time increase morbidity and may lead to high mortality rates, thus becoming a problem of great personal, family and socio-health significance (Tena & Serrano, 2002; Stratton, 2012). The prevalence of malnutrition ranges between 1% and 6% in the free-living elderly, but the risk of suffering it may reach up to 60% (Tena & Serrano, 2002; Guigoz, 2006; Kaiser et al., 2010; Sánchez-Muñoz et al., 2013). Its incidence is high in both underdeveloped and more developed countries and it is a challenge for society in terms of the sustainability of its economic resources and health systems (Stratton, 2012; García de Lorenzo, Álvarez & De Man, 2012).

Currently it is estimated that the majority of the elderly (96.2%) are free living and want to remain at their home or at a relative’s home as long as possible (Iglesias de Ussell & López Doblas, 2014). The 2012 IMSERSO report (Madrigal Muñoz, 2014) estimates that over 96% of the elderly in the Valencia Region live at home. One study from the 3rd Plenufar Plan (Nutrition Education by Pharmacists Plan) found that 3.7% of these free-living elderly are malnourished and 22.2% at risk of becoming so (Plenufar III, 2006). Furthermore, in a previous study (Montejano et al., 2013), we found that 23.3% of this group in the province of Valencia are at risk of malnutrition.

Given the forcast of experts this population group is set to increase, so it would be advisable and effective to set up health programmes in primary healthcare which would enable early identification of nutritional risk, thus avoiding malnutrition and its possible consequences. Only through adequate, quick and inexpensive nutritional assessment can some nutritional problems be resolved or at least be alleviated (Tena & Serrano, 2002; Cuesta, 2007).

Various scales have been used to perform a quick and first level nutritional assessment, and the Mini Nutritional Assessment (MNA) is one of the most used and recommended by experts in the elderly in all areas. One of its main advantages is the identification of malnutrition risk before clinical alterations onset and thus does not require laboratory tests. Since 1994 and after 20 years of development and use, the MNA scale has become established as one of the most commonly used tools by researchers for first-level nutritional status assessment of the elderly, irrespective of where they live. As it is easy to use, inexpensive and reliable it has become part of the clinical practice of health professionals and a valid and essential element in comprehensive geriatric assessment (Guigoz, Vellas & Garry, 1994; Cuesta, 2007; Bauer et al., 2008; Kaiser et al., 2011; Salvà, 2012).

This internationally validated and used scale has a short/screening version, the Mini Nutritional Assessment Short Form (MNA-SF), which was developed and validated in 2001 (Rubenstein et al., 2001). With the addition of this short form, the nutritional assessment occurs in two stages, the first in order to identify people at risk of malnutrition and a second one in which these people are assessed with the full MNA scale (Rubenstein et al., 2001; Bauer et al., 2008; Salvà, 2012). The MNA-SF was revised in 2009 and a malnutrition status cut-off point was added in order to classify the elderly into three groups (normal nutrition status, risk of malnutrition and malnutrition) matching these three categories in the full version. Another new feature is that Body Mass Index (BMI) can be replaced by Calf Circumference (CC) in the elderly where there is difficulty in measuring height and weight. If the people assessed get a score lower than 12 on these short scales, it is advisable to complete the full MNA scale to avoid diagnostic accuracy errors (Kaiser et al., 2009). Furthermore, when the full MNA scale identifies malnutrition or the risk of malnutrition, further examination should be performed at a second level including a dietary history to determine biochemical, immunological and anthropometric parameters since, as noted by some authors and international organisations, there is no single parameter by itself which allows nutritional diagnosis (Calderón et al., 2010; Durán Alert et al., 2012). The two short versions of the scale present good diagnostic prediction compared to the full scale (Kaiser et al., 2009; Kaiser et al., 2011; García-Meseguer & Serrano-Urrea, 2013; Lilamand et al., 2015; Lera et al., 2016) but there are many more validation studies for the original short scale (BMI-MNA-SF) than the short one revised in 2009 (CC-MNA-SF).

Recently, the MNA scale has been used as the gold standard to develop other short nutritional assessment questionnaires for free-living elderly. Malek Mahdavi et al. (2015) have drawn up a scale with nine questions, all included in the MNA (clinical status, dietary assessment and self-perception of health status and nutrition together with mid-arm and calf circumference measurements without including body mass index). In Taiwan, Tsai, Chang & Wang (2013) developed new tools based on the MNA, combining some items and changing the BMI, CC and MNA cut-off points to adapt them to their population characteristics. Gutiérrez-Gómez et al. (2015), after assessing a sample of elderly women with MNA and obtaining factors associated with malnutrition, designed and validated a questionnaire with six items (physical activity, diabetes, hypertension, dentition, psychological problems and living with family). All of them have obtained good diagnostic accuracy results compared to the MNA scale.

Some authors (Phillips et al., 2010; De la Montaña & Miguez, 2011) have compared the MNA with other nutritional screening instruments in the free-living elderly and classified MNA as one of the best tools for this group due to its high sensitivity, reliability and validity. However, there are few studies comparing the validity and predictive capacity of the BMI-MNA-SF and CC-MNA-SF scales with respect to the full MNA scale in exclusively free-living elderly samples. Most studies use heterogeneous samples with dependent or non-dependent elderly living in nursing homes, hospitals and the community. In this study we sought to evaluate the sensitivity, specificity and predictive ability of this scale in both its original (BMI-MNA-SF) and modified (CC-MNA-SF) versions against the full MNA scale in a previously studied free-living and non-dependent elderly population in Spain (Montejano, 2012; Montejano et al., 2013).

## Materials and Methods

### Design and sample

We designed a cross-sectional descriptive study including 660 people of both sexes aged 65 or over, free-living and residing in the province of Valencia. The study was conducted between October 2008 and November 2009. We collected data in twelve community centres for the elderly across the province of Valencia, which were selected using stratified sampling by blocks to ensure the various geographical settings of the province were represented. We chose a sample size that would ensure an estimate of the proportion of risk or malnutrition with an accuracy of 5% and a power of 80% for the tests performed, considering an estimated population prevalence of 35%.

Inclusion criteria for participating in the study were being aged 65 or over, living at home, having resided in the province of Valencia for over one year, regularly attending community centres and voluntarily wanting to take part in the study.

### Study population

National Institute of Statistics (INE) data show that in 2009, people aged 65 and over registered in the province of Valencia accounted for 15.9% of its total population. 95% of them lived in the community (in free-living) and were regular users of socio-health centres. Valencia’s health system is free and universal and the province of study has 11 health departments with 142 health centres and 281 auxiliary offices distributed throughout its territory. They draw up health plans for the elderly to improve their healthy habits and lifestyles (Montejano, 2012).

### Study variables

We used three scales, the full MNA and its two short versions (BMI-MNA-SF and CC-MNA-SF), for the nutritional assessment of the participants. We used the full MNA as the gold standard for nutritional assessment.

The full MNA scale has 18 items, provides a maximum possible overall assessment of 30 points and its nutritional categories are normal nutritional status (24–30 points), risk of malnutrition (17–23.5 points) and malnutrition (<17 points) (Guigoz, Vellas & Garry, 1994). The MNA-SF has two versions depending on the anthropometric measurement used. The BMI-MNA-SF contains the first six items of the full scale plus Body Mass Index while the CC-MNA-SF includes Calf Circumference instead of BMI. In both scales the maximum possible score is 14 points and, based on this score, there are three nutritional categories matching the full scale, namely normal nutritional status (12–14 points), risk of malnutrition (8–11 points) and malnutrition (0–7 points). According to the authors, a score lower than 12 suggests possible malnutrition and makes it advisable to fill in the MNA to get a more precise evaluation of nutritional status (Kaiser et al., 2009).

The data to complete the test was obtained from personal interviews conducted in the community centres by nurses who had been trained beforehand for that purpose. The instructions in the guide to completing the MNA test (Nestle Nutrition Institute, 2008) were followed. The anthropometric parameters in the scale, Mid-Arm Circumference (MAC), Calf Circumference (CC) and Body Mass Index (BMI), were gathered.

CC and MAC were measured with a SECA 201 tape measure (range 0–205 cm and 1 mm accuracy). MAC was measured in the non-dominant arm at the midpoint between the acromion and the olecranon with the arm hanging down and parallel to the body. CC was measured in the calf area where the circumference is greatest with the person sitting down, resting their foot on the floor and their knee bent at 90°.

Weight and height were measured with an ASIMED MB201 scale and height rod with an accuracy of 100 g for weight (range 0.1–150 kg) and an accuracy of 0.5 cm for height (maximum capacity 200 cm). Weight was measured with the subject barefoot and wearing light clothing to the nearest 0.1 kg. Height was measured with the subject standing barefoot with heels together, arms at their sides, legs straight, shoulders relaxed and head aligned in the Frankfort horizontal plane with heels, buttocks, shoulder blades and the back of the head supported against the height rod. Measurements were recorded in centimetres. Body mass index was calculated as weight (kg) divided by height^2^ (m) (BMI = weight (kg)/height^2^ (m)).

### Statistical analysis

We conducted descriptive analysis of the variables of interest. We used means, standard deviations, medians and ranges to describe quantitative variables and the Kolmogorov–Smirnov test to check normality. We used proportions to describe qualitative variables and analysed linear association between the scores obtained in the short forms and the full version of the MNA test by Spearman’s test for correlations. Based on the categories “malnutrition”, “risk of malnutrition” and “normal nutritional status” we obtained Cohen’s kappa index to quantify agreement greater than that expected by chance between the MNA-SFs and full MNA classification. We obtained the confidence intervals for the estimated kappa values using the jackknife method which does not require normality for the variables (Efron & Tibshirani, 1993; Abraira & Pérez de Vargas, 1999). We performed a kappa homogeneity test to compare of the kappa index (Fleiss, 1981).

Based on the categories “malnutrition and risk of malnutrition” vs. “normal nutrition status” and using the full MNA as the gold standard, we calculated sensitivity, specificity and predictive values in order to assess the diagnostic accuracy of the short forms of the MNA test. Additionally, we performed a non-parametric estimation of the ROC curve to evaluate the discriminatory power of the BMI-MNA-SF and CC-MNA-SF independently of the cut-off values. We used graphic representation and the Hanley and McNeil test to compare the diagnostic power of the two MNA-SFs. We performed statistical analyses with the SPSS (Statistical Package for Social Sciences) version 20.0 for Windows XP (SPSS Inc., Chicago, IL, USA) and Epidat 4.2 (Epidat, 2016).

### Ethical and legal aspects

The proposal was reviewed by the Doctoral Committee of the Department of Nursing, Faculty of Health Sciences. The study has been approved by the Ethics Committee at the University of Alicante (File UA 2016-12-21).

A formal written request to obtain permission for the study was made to the management teams at the community centres for the elderly included in it. All study subjects were asked to take part on a voluntary basis. They were properly informed about the study and their anonymity was guaranteed. Legal requirements and guidelines for good clinical practice and the World Medical Association Declaration of Helsinki on Ethical Principles for Medical Research involving Human Subjects (revised in October 2008) were met.

## Results

The basic characteristics of the study population are shown in Table 1. Of the 660 subjects studied, 319 were men (48.3%) and 341 (51.7%) women with a mean age of 74.3 years (SD = 6.6); 54% were aged between 65 and 74 and most of them live with someone else (72.7%).

**Table 1:** Basic characteristics of the Study Population.

**Characteristics**	
**Men,***n* (%)	319 (48, 3)
**Women,***n* (%)	341 (51, 7)
**Age groups**	
65–74 years, *n* (%)	357 (54)
75–84 years, *n* (%)	256 (38, 8)
≥85 years, *n* (%)	47 (7, 1)
**Living alone,***n* (%)	180 (27, 3)
**Live together,***n* (%)	480 (72, 7)
**Body mass index (kg/m**^**2**^), mean (SD^a^)	29 (4, 1)
**Mid-arm circumference (cm),** mean (SD)	29, 6 (3, 1)
**Calf circumference (cm),** mean (SD)	36, 1 (3, 3)

**Notes.**

a Standard deviation.

**Figure 1:**
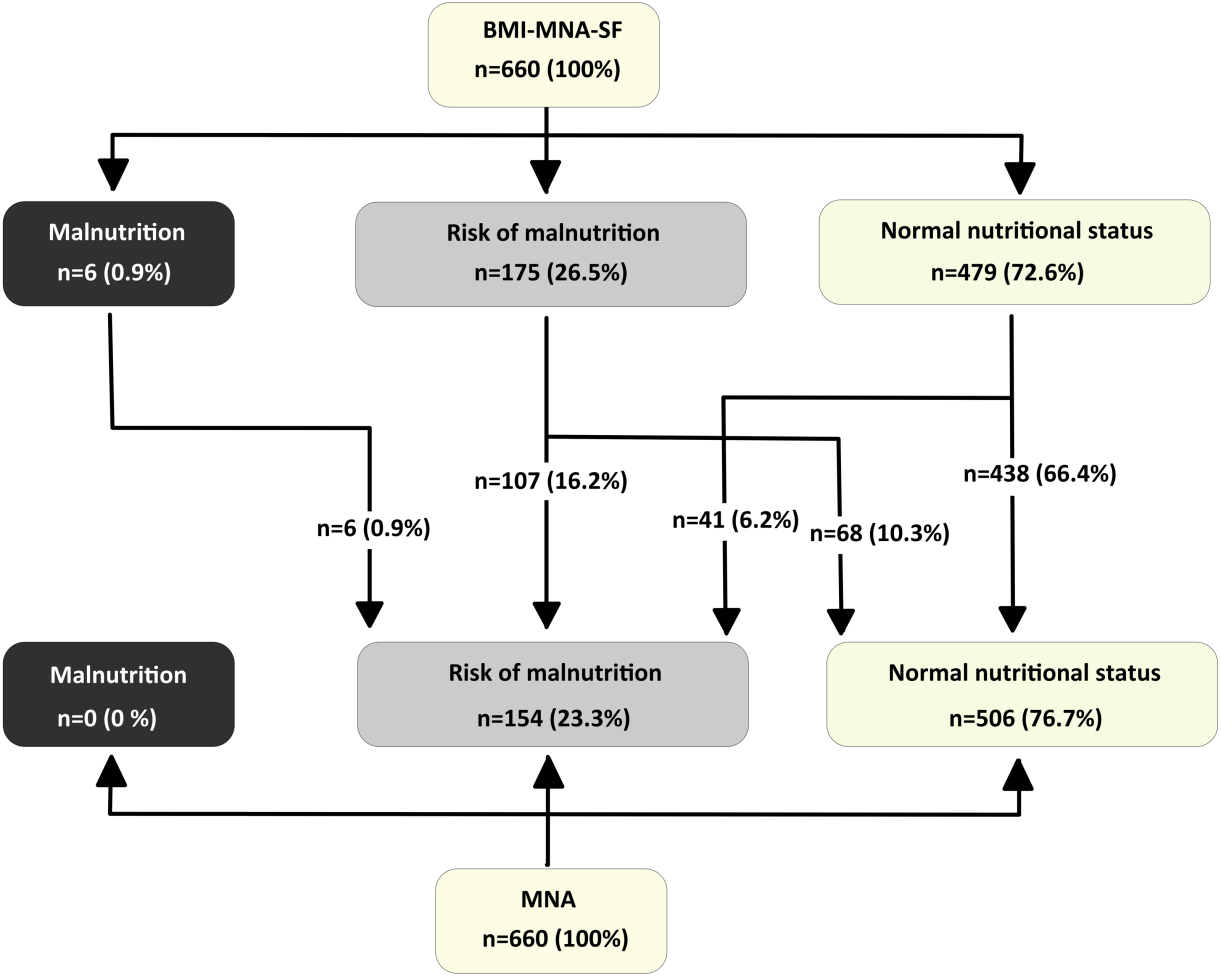
Distribution of Nutritional Status in elderly people-participants for the BMI-MNA-SF scale and the full MNA scale.

**Figure 2:**
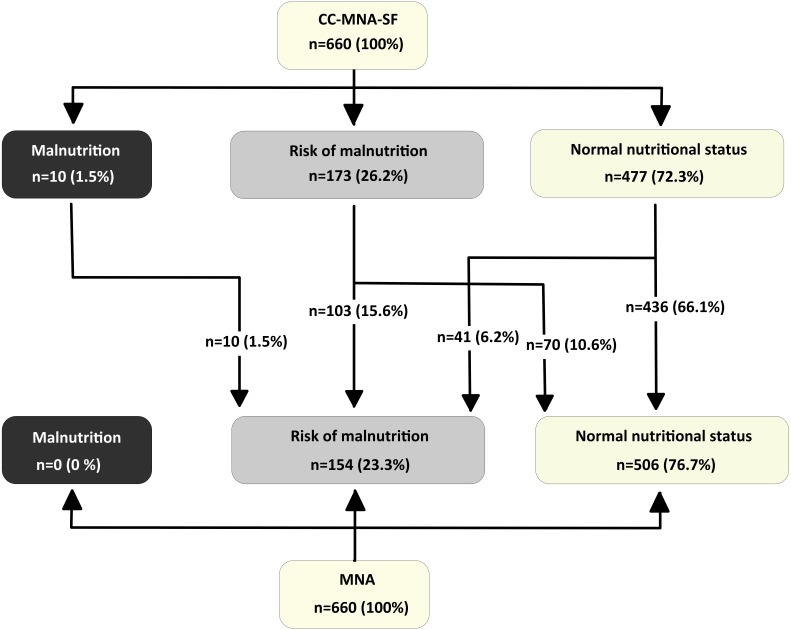
Distribution of Nutritional Status in elderly people-participants for the CC-MNA-SF scale and the full MNA scale.

The mean scores we obtained in the three scales are high and are very similar in the two short scales. Out of a possible 14 points offered by both versions, the elderly respondents achieved an average score of 12.4 points (SD = 1.6) on the BMI-MNA-SF scale and 12.3 points (SD = 1.7) on the CC-MNA-SF scale. On the full version of the MNA, out of a possible 30 points they achieved a mean score of 25.3 points (SD = 2.4). Figures 1 and 2 show the categories of diagnostic classification based on the nutritional status of respondents for the full version and the two short versions of the MNA. They are expressed as frequencies and percentages. Categorising the three nutritional assessment possibilities offered by the MNA according to the total score shows that of the 660 elderly assessed, 154 (23.3%) were at risk of malnutrition; malnutrition itself was not detected in any of them. There are similarities in the classification using the two short scales; with the BMI-MNA-SF, 175 (26.5%) were at risk of malnutrition and six (0.9%) were malnourished, while with the CC-MNA-SF scale 173 (26.2%) were at risk of malnutrition and 10 (1.5%) were malnourished. Following the instructions of the scale, all those people assessed with the two short versions who did not obtain scores above 11 points had to complete the MNA test in order to clarify their nutritional status (Kaiser et al., 2009). With the BMI-MNA-SF scale, we found 181 (27.4%) people who had not passed this score and with the CC-MNA-SF we found a total of 183 (27.7%), all of whom therefore had to complete the MNA.

The categorical classification given by the short versions shows that of the 479 persons classified with normal nutritional status by the BMI-MNA-SF and the 477 people classified with normal nutritional status by the CC-MNA-SF, in both cases there were 41 people at risk of malnutrition according to the full version of the MNA. In other words, had we not completed the full version of the scale, these 41 people (6.2% of the total sample) would have passed both screening tests without being considered at risk of malnutrition when in fact they were (Figs. 1 and 2).

Table 2 shows the comparative study we performed on the sample of free-living elderly residents in the province of Valencia, the results obtained with the MNA-SF short versions and the results obtained with the full MNA. To get an overall view we obtained different measures of association and agreement. Spearman’s rank correlation coefficients indicate a high association between the full MNA score and the MNA-SFs scores (BMI-MNA-SF: *ρ* =0.78 *p* < 0.001; CC-MNA-SF: *ρ* = 0.78*p* < 0.001). There is also very high correlation between the two MNA-SFs (*ρ* = 0.96*p* < 0, 001).

**Table 2:** Comparative study of measurements of agreement between MNA-SFs and full MNA.

	BMI-MNA-SF	CC-MNA-SF	*p*-valor
**Scores**			
**Spearman’s correlation**	0,78 (0,75–0,81)	0,78 (0,76–0,80)	
**Classification in categories**			
3 categories **(malnutrition,risk of malnutrition, normal nutritional status)**			
Cohen’s κ (95% CI)	0,54 (0,47–0,62)	0,52 (0,45–0,60)	0,697
2 categories **(malnutrition-risk of malnutrition vs normal nutritional status)**			
Cohen’s κ (95% CI)	0,57 (0,49–0,64)	0,56 (0,49–0,63)	0.907
**Diagnostic test (malnutrition-risk of malnutrition vs normal nutritional status)**			
AUC (95% CI)			
Sensitivity	0,88 (0,85–0,91)	0,87 (0,84–0,90)	0,581
Specificity	73,4%	73,4%	
PPV^a^	86,6%	86,2%	
NPV^b^	62,4%	61,7%	
Diagnostic efectiveness	91,4%	91,4%	
(% correctly classified)	83,5%	83,2%	

**Notes.**

a Positive predictive value.

b Negative predictive value.

c Area under the curve.

Since the point of the MNA is classifying free-living elderly in the three nutritional statuses (normal, at risk and malnutrition), we completed this coefficient with a measure of agreement. The kappa coefficient indicates the agreement over and above the amount we would expect by chance. When interpreting the values of this coefficient according to the Landis & Koch (1997) scale and comparing them with the values obtained in other studies, it should be borne in mind that they are influenced by the prevalence of the disease in the population considered. In our study the kappa values obtained for the MNA-SFs (BMI-MNA-SF: κ = 0.54*p* < 0.001; CC-MNA-SF: κ = 0.52*p* < 0.001) indicate moderate agreement with the full MNA according to Landis & Koch (1997) classification and there is very good agreement between the BMI-MNA-SF and the CC-MNA-SF (κ = 0.88*p* < 0.001). If we compare the BMI-MNA-SF and CC-MNA-SF kappa coefficients using a kappa homogeneity test, we do not obtain any significant difference (*p* = 0.697).

**Figure 3:**
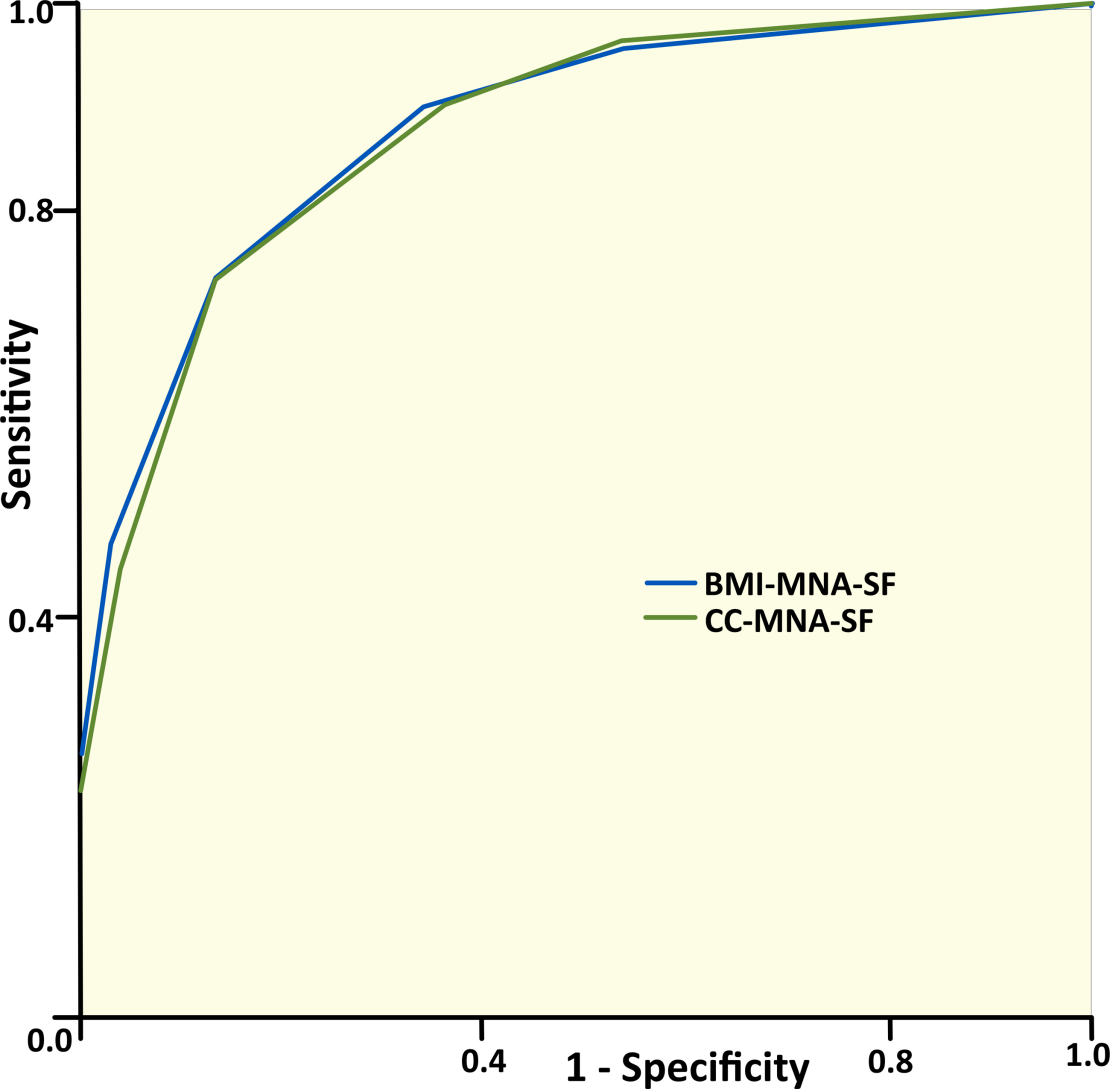
ROC curves for the studied sample applying the BMI-MNA-SF and the CC-MNA-SF as compared with the full MNA. Area under the curve for BMI-MNA-SF = 0.88 (95% CI [0.85–0.91]). Area under the curve for CC-MNA-SF = 0.87 (95% CI [0.84–0.90]).

In order to determine the ability of both MNA-SFs to identify subjects not requiring any nutritional intervention, we considered the dichotomised categorisation in the full MNA and the MNA-SFs as “risk of malnutrition” vs. “normal nutritional status”. Areas under the ROC curves using the full MNA as the gold standard reached high values (BMI-MNA-SF: AUC =0.88*p* < 0.001; CC-MNA-SF AUC =0.87*p* < 0.001) (Fig. 3). We do not obtain a significant difference if we compare the diagnostic accuracy of the two short tests (*p* = 0.581). Considering the full MNA as the gold standard, the sensitivity obtained in the MNA-SFs is similar (73.4%) and the specificity is slightly higher in the BMI-MNA-SF than in the CC-MNA-SF (86.6% and 86.2%). Although prevalence does not affect sensitivity or specificity, the sensitivity value will decrease if the degree of malnutrition presented by the elderly is not very high. Given that the free-living elderly population resides at home, the prevalence of malnutrition is expected to be low when compared to hospitalised populations.

The predictive values contained in Table 2 (PPV, NPV and diagnostic effectiveness) will be affected by the prevalence of malnutrition in the population, with PPV going down in populations with low prevalence.

## Discussion

### Summary

In this study, we found that the BMI-MNA-SF and CC-MNA-SF scales have adequate predictive accuracy with respect to the MNA scale in non-institutionalized elderly. They showed high sensitivity and accuracy. We also obtained a moderate agreement between MNA and the two versions of the MNA-SF for identifying subjects with risk of malnutrition. In both cases, the overestimation of the nutritional status is only 6.2% compared to the MNA which is considered the gold standard. Malnutrition was not detected in the sample considered in our study (non-institutionalized elderly attending day care centres) and the percentage of adults at risk of malnutrition was 23.3%.

### Strengths and limitations

One limitation of our study is that the sample is not representative of the whole group of free-living elderly, as people with disabilities have been excluded.

The strength of our study is that diagnostic accuracy tests of the CC-MNA-SF scale for an exclusively free-living elderly sample had not previously been conducted in Spain.

### Comparison with existing literature

Due to the high influence of the disease prevalence and its severity on statistical measures used to compare diagnostic tests (Burgueño, García-Bastos & Gónzalez-Buitrago, 1995), some studies have been conducted to evaluate the MNA test and MNA short versions in different populations. The information provided by these studies is really interesting for the specialist may take right decisions based on the characteristics of the population on which the tests are being performed. As we mentioned above, few studies have been done up to the moment in non-institutionalized elderly populations and especially on the use of CC-MNA-SF on these type of populations, where it may have a very useful result.

We have performed an exhaustive revision of recently studies performed on the MNA, BMI-MNA-SF or CC-MNA-SF in non-institutionalized elderly populations or in mixed populations. At the international level, we have found one study performed in Germany and Italy with 675 elderly people divided into three groups: community-dwelling older people, nursing homes residents and patients in geriatric rehabilitation (Kaiser et al., 2011), concerning the diagnostic precision of the BMI-MNA-SF and CC-MNA-SF scale, whereas the scale MNA as gold standard. The prevalence rates in institutionalized (malnutrition 18.2% risk 41.9%) or in rehabilitation (malnutrition 40.8% risk 45.9%) older patients was very high. The prevalence in non-institutionalized older adults, who were recruited by an article in press and went to take the test on its own, is low (0% malnutrition risk 11%). The level of agreement between the two short forms and the MNA, was good in institutionalized or in rehabilitation older patients and was however moderated in non-institutionalized older adults, as in our study, being the coefficients kappa slightly lower in the CC-MNA-SF than in the BMI-MNA-SF in the three groups. The comment by the authors, in the discussion, that the moderate agreement obtained in non-institutionalized older adults is due to the low prevalence rates.

Lera et al. (2016) carried out a comparative study between the three scales with free-living elderly, aged over 60, in five cities in Latin America (São Paulo, Santiago, Havana, Mexico and Montevideo). They conducted a random sampling, calling people by phone and visiting them in their homes. The overall prevalence of malnutrition was 3.42% and risk of malnutrition 25%. The level of agreement among the three scales is good in the mentioned cities, varying the kappa coefficient based on the different level of prevalence observed in each city. We consider that the level of agreement is a little bit greater than the obtained in our study, due to the higher prevalence of malnutrition on the five cities considered. The study on the sensitivity and specificity of the CC-MNA-SF was conducted considering as gold-standard BMI-MNA-SF, so the results are not comparable with the values obtained in our study.

In Spain we found two studies (Cuervo et al., 2008; De la Montaña & Miguez, 2011) where the sample characteristics are similar to ours. They only carried out tests for the validity and diagnostic accuracy of the BMI-MNA-SF scale with respect to the MNA.

Cuervo et al. (2008) conducted a study on malnutrition in Spain with patients not institutionalized using the MNA and the BMI-MNA-SF, without considering CC-MNA-SF. The study included 22,007 patients 65 years or older. The patients were recruited in pharmacies, where they bought their medicines. The prevalence was slightly higher than ours, as in Lera’s et alt. study (malnutrition 4.3% and risk 25.4%) possibly since were recruited in pharmacies while they were buying medicines. The level of agreement between MNA and BMI-MNA-SF on this study is good, due to the same reasons we have mentioned previously.

De la Montaña & Miguez (2011) conducted the study in the north-western of Spain among elderly living in their homes and needing social care, to assess the suitability of the BMI-MNA-SF test for this population. The sample size was 728 and the prevalence was very high (12.5% malnutrition and 57% at risk). Both the sensitivity and specificity are higher than ours, which may be due to the severity of malnutrition in patients based in this environment. The PPV was very high and the NPV was very low, possibly due to the high prevalence, opposite to the results of our study where the PPV is low and the NPV is high, due to the low prevalence. The global predictive value is similar.

The only study we have found in Spain, where the CC-MNA-SF is compared to the MNA test is the one by García-Meseguer & Serrano-Urrea (2013), developed in Albacete in patients institutionalized with a high prevalence (2.8% malnutrition and 37.3% at risk). On this study was achieved a good agreement between BMI-MNA-SF and MNA, a moderate agreement between CC-MNA-SF and MNA and higher values for specificity and sensitivity, possibly associated with higher level of malnutrition.

It may be observed in former studies, that when we are working on a population where de prevalence of malnutrition is low, as in our case, the existence of an imbalance between the percentage of elderly people in a normal nutritional state and the percentage of elderly people at risk or malnourished, does the random agreement be greater and the kappa coefficient has smaller values. Also, the low prevalence of malnutrition has negative influence on the predictive positive value and positive on the negative predictive value. On the other hand, even though prevalence does not have a direct influence on the sensitivity and specificity of diagnostic test, these values are influenced by the level of malnutrition, because of the difficulty of detecting elderly people with mild level of malnutrition.

This justifies the importance of doing studies on these diagnostic tests on different populations to be able to get proper decisions based on the results obtained. On the few studies done up to this moment, in populations with a malnutrition prevalence and a level of malnutrition similar to that of the patients analysed on this study, results are similar to ours. Also, we think this is one of the first studies where the CC-MNA-SF test has been analysed with non-institutionalized elders in Spain.

### Implications for research and/or practice

Bearing in mind that the free-living elderly population is growing and that inexpensive, quick and efficient nutritional assessment tools are needed for use in primary healthcare settings, we suggest that the CC-MNA-SF scale is a useful screening tool and a valid alternative to the BMI-MNA-SF, although the characteristics of the population must always be taken into account to make the right decisions based on the MNA-SF scales. Since malnutrition prevalence is usually not very high in this type of population, the level of agreement between the BMI-MNA-SF, CC-MNA-SF and the MNA will be moderate. On the other hand, given that malnutrition is not usually present at an advanced stage in free-living elderly, the sensitivity of short-forms scales also may be affected. Therefore, when the CC-MNA-SF results are not in accordance with other additional information and the doctor is in doubt, the proper decision will be to complete the full MNA or perform additional examinations.

## Conclusions

To sum up, in our study, both versions of the MNA-SF have high predictive ability to identify nutritional risk in free-living elderly people. In both cases there was an overestimation of their nutritional status of only 6.2%. The new version (CC-MNA-SF) is easier to use as the BMI parameter can be replaced by CC in primary care centres, taking less time and being less costly, as it does not require scales and a large equipment to measure weight and height.

However, more studies would be needed in order to better understand their discriminatory capacity in non-institutionalised elderly populations. It would also be useful to study the problems to identify potential malnutrition in this type of population, since its degree of malnutrition is low and also to investigate the possibility of raising the CC-MNA-SF cut-off point for this group.

##  Supplemental Information

10.7717/peerj.3345/supp-1 Supplemental Information 1 Dataset

## Additional Information and Declarations

### Data Availability

The following information was supplied regarding data availability: The raw data has been supplied as Data S1.

## References

[ref-1] Abraira V, Pérez de Vargas A (1999). Generalization of the kappa coefficient for ordinal categorical data, multiple observers and incomplete designs. Qüestiió.

[ref-2] Bauer JM, Kaiser MJ, Anthony P, Guigoz Y, Sieber CC (2008). The mini nutritional assessment-its history, today’s practice, and future perspectives. Nutrition in Clinical Practice.

[ref-3] Burgueño MJ, García-Bastos JL, Gónzalez-Buitrago (1995). Las curvas ROC en la evolución de las pruebas diagnósticas. Medicina Clínica.

[ref-4] Calderón ME, Ibarra F, García J, Gómez C, Rodríguez-Orozco AR (2010). Evaluación nutricional comparada del adulto mayor en consultas de medicina familiar. Nutrición Hospitalaria.

[ref-5] Cuervo M, García A, Ansorena D, Sánchez-Villegas A, Martínez-González MA, Astiasarán I, Martínez JA (2008). Nutritional assessment interpretation on 22 007 Spanish community-dwelling elders through the mini nutritional assessment test. Public Health Nutrition.

[ref-6] Cuesta F, SENPE y SEGG (2007). Cuestionarios estructurados de valoración del riesgo nutricional. Valoración nutricional en el anciano. Recomendaciones prácticas de los expertos en geriatría y nutrición.

[ref-7] De la Montaña J, Miguez M (2011). Suitability of the short-form mini nutritional assessment in free-living elderly people in the northwest of Spain. The Journal Nutrition Health and Aging.

[ref-8] Durán Alert P, Milá Villarroel R, Formiga F, Virgili Casas, Vilarasau Farré C (2012). Assessing risk screening methods of malnutrition in geriatric patients; mini nutritional assessment (MNA) versus geriatric nutritional risk index (GNRI). Nutrición Hospitalaria.

[ref-9] Efron B, Tibshirani RJ (1993). An introduction to the bootstrap.

[ref-10] Epidat (2016). Consellería de Sanidade de Xunta de Galicia, España; OPS-OMS.

[ref-11] Fleiss JL (1981). Statistical methods for rates and proportions.

[ref-12] García de Lorenzo A, Álvarez J, De Man F (2012). Envejecimiento y desnutrición; un reto para la sostenibilidad del SNS; conclusiones del IX Foro de Debate Abbott-SENPE. Nutrición Hospitalaria.

[ref-13] García-Meseguer MJ, Serrano-Urrea R (2013). Validation of the revised mini nutritional assessment short-forms in nursing homes in Spain. The Journal Nutrition Health and Aging.

[ref-14] Guigoz Y (2006). The mini nutritional assesment (MNA) review of the literature- what does it tell us?. The Journal Nutrition Health and Aging.

[ref-15] Guigoz Y, Vellas B, Garry PJ (1994). Mini nutritional assessment: a practical assessment tool for grading the nutritional state of elderly patients. Facts and Research in Gerontology.

[ref-16] Gutiérrez-Gómez T, Cortés E, Palazón-Bru A, Peñarrieta-de Cordova I, Gil-Guillen VF, Ferrer-Diego RM (2015). Six simple questions to detect malnutrition or malnutrition risk in elderly women. PeerJ.

[ref-17] Iglesias de Ussell J, López Doblas J (2014). Participación social, formas de vida, relaciones sociales y la forma de envejecer. Ministerio de sanidad, servicios sociales e igualdad. Instituto de Mayores y Servicios Sociales (IMSERSO) (Ed. de la serie). Informe 2012 las personas mayores en España. Datos estadísticos estatales y por comunidades autónomas.

[ref-18] Kaiser MJ, Bauer JM, Rämsch C, Uter W, Guigoz Y, Cederholm T, Thomas DR, Anthony P, Charlton KE, Maggio M, Tsai AC, Grathwohl D, Vellas B, Sieber CC, MNA-International Group (2009). Validation of the mini nutritional assessment short-form (MNA-SF): a practical tool for identification of nutritional status. The Journal Nutrition Health and Aging.

[ref-19] Kaiser MJ, Bauer JM, Rämsch C, Uter W, Guigoz Y, Cederholm T, Thomas DR, Anthony PS, Charlton KE, Maggio M, Tsai AC, Vellas B, Sieber CC (2010). Frequency of malnutrition in older adults: a multinational perspective using the mini nutritional assessment. Journal of the American Geriatrics Society.

[ref-20] Kaiser MJ, Bauer JM, Uter W, Donini MD, Stange I, volkert D, Diekmann R, Drey M, Bollwein J, Tempera S, Guerra MD, Ricciardi LM, Sieber CC (2011). Prospective validation of the modified mini nutritional assessment short-forms in the community, nursing home, and rehabilitation setting. Journal of the American Geriatrics Society.

[ref-21] Landis JR, Koch GG (1997). The preasurement of observer agreement for categorical data. Biometrics.

[ref-22] Lera L, Sánchez H, Ángel B, Albala C (2016). Mini nutritional assessment short-form: validation in five latin american cities. SABE study. The Journal Nutrition Health and Aging.

[ref-23] Lilamand M, Kelaiditti E, Cesari M, Raynaud-Simon A, Ghisolfo A, Guyonnet S, Vellas B, Abellan Van Kan G and Toulouse Frailty Platform Team (2015). Validation of the mini nutritional assessment-short form in a population of frail elders without disability. Analysis of the toulouse frailty platform population in 2013. The Journal Nutrition Health and Aging.

[ref-24] Madrigal Muñoz A (2014). Servicios sociales dirigidos a personas mayores en España. Diciembre 2011. Ministerio de sanidad, servicios sociales e igualdad. Instituto de Mayores y Servicios Sociales IMSERSO (Ed. de la serie). Informe 2012. Las personas mayores en España. Datos estadísticos estatales y por comunidades autónomas.

[ref-25] Malek Mahdavi A, Mahdavi R, Lotfipour M, Asghari Jafarabadi MA, Faramarzi E (2015). Evaluation of the Iranian mini nutritional assessment short-form in community-dwelling elderly. Health Promotion Perspectives.

[ref-26] Montejano R (2012). Evaluación de riesgo nutricional y de factores asociados en adultos mayores no Institucionalizados en la provincia de Valencia. Thesis.

[ref-27] Montejano R, Ferrer RM, Clemente G, Martínez-Alzamora N (2013). Estudio del riesgo nutricional en adultos mayores autónomos no institucionalizados. Nutrición Hospitalaria.

[ref-28] Nestle Nutrition Institute (NNI) (2008). Guía para la cumplimentación del MNA. http://www.mna-elderly.com/forms/mna_guide_spanish.pdf.

[ref-29] Phillips MB, Foley AL, Barnard R, Isenring EA, Miller MD (2010). Nutritional screening in community-dwelling older adults: a systematic literature review. Asia Pacific Journal of Clinical Nutrition.

[ref-30] Plan de educación nutricional por el farmacéutico (Plenufar III) (2006). Educación nutricional a las personas mayores. Vocalía Nacional de Alimentación. Consejo General de Colegios Oficiales de Farmacéuticos.

[ref-31] Rubenstein LZ, Harper JO, Salvà A, Guigoz Y, Vellas B (2001). Screening for undernutrition in geriatric practice: developing the short-form mini-nutritional assessment (MNA-SF). Journal of Gerontology: Medical Sciences.

[ref-32] Salvà A (2012). El mini nutritional assessment. Veinte años de desarrollo ayudando a la valoración nutricional. Revista Española de Geriatría y Gerontología.

[ref-33] Sánchez-Muñoz LA, Serrano-Monte A, Pita Álvarez J, Jauset Alcalá C (2013). Valoración nutricional con Mini Nutritional Assessment. Medicina Clínica.

[ref-34] Stratton RJ (2012). Clinical and economic effects of managing malnutrition. Nutrición Hospitalaria.

[ref-35] Tena MC, Serrano P, A Salgado and F, Guillén and I, Ruipérez (2002). Malnutrición en el anciano. Manual de Geriatría.

[ref-36] Tsai AC, Chang TL, Wang JY (2013). Short-form mini-nutritional assessment with either BMI or calf circumference is effective in rating the nutritional status of elderly Taiwanese–results of a national cohort study. British Journal of Nutrition.

[ref-37] Vidal Domínguez MJ, Fernández Portela J (2014). Indicadores demográficos. Ministerio de Sanidad, Servicios Sociales e Igualdad. Instituto de Mayores y Servicios Sociales (IMSERSO) (Ed. de la serie). Informe 2012. Las personas mayores en España. Datos estadísticos estatales y por Comunidades autónomas.

